# Supplementary material for: Protective Effect of Treated Olive Mill Wastewater on Target Bacteria and Mitochondrial Voltage-Dependent Anion-Selective Channel 1
Source: Antioxidants (Basel). 2023 Jan 30;12(2):322. doi: 10.3390/antiox12020322 (PMC9951878; doi:10.3390/antiox12020322)
Supplement: Supplementary file 1 [file antioxidants-12-00322-s001.zip › antioxidants-2159361-supplementary-for conversion.pdf]

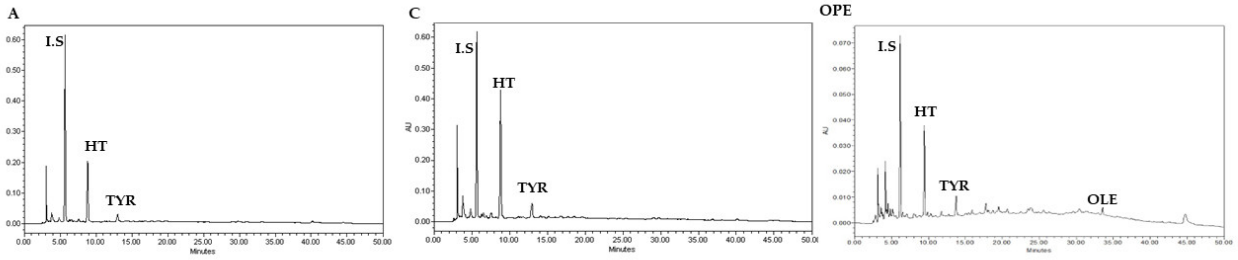

Figure S1. HPLC Chromatogram of phenols of A, C and OPE sample detected at 280 nm.

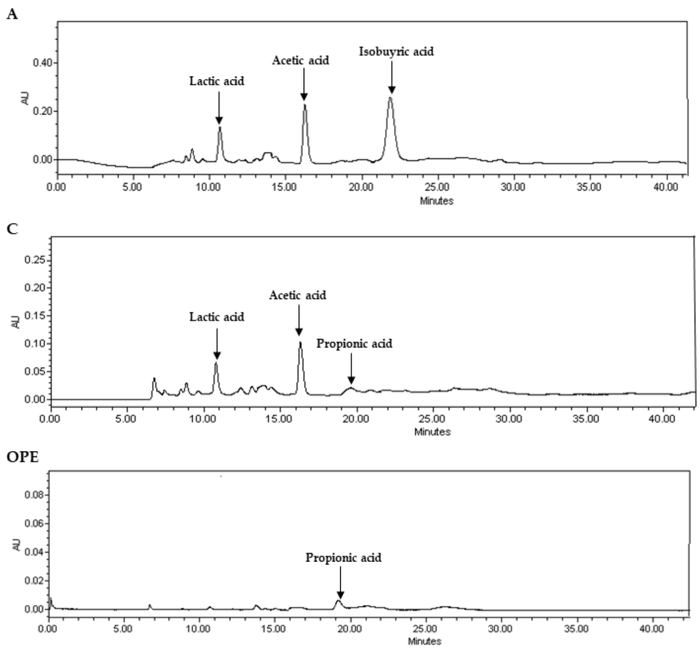

Figure S2. HPLC Chromatogram of organic acids of A, C and OPE sample detected at 210 nm.

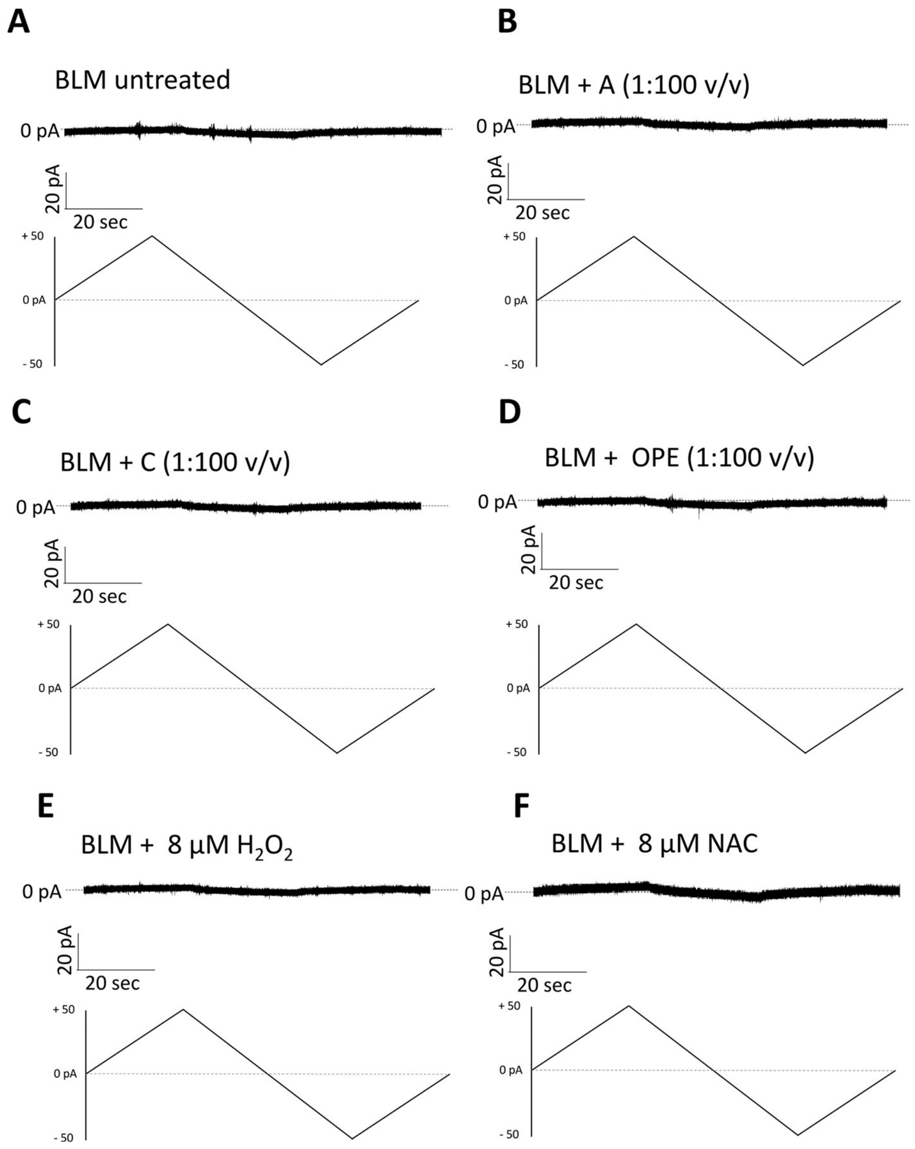

Figure S3. Evaluation of the effect of tested samples, hydrogen peroxide and N-acetyl cysteine (NAC) on membrane stability by triangular voltage ramps. Samples A (B), C (C) and OPE (D), H_2O_2 (E) and NAC (F) were added to both sides of a DiphPC bilayer at a final concentration of 1:100 (v/v) and 8 μM, respectively, to exclude any interferent effect with the lipid membrane. In each experiment, the stability of the current signal was monitored upon application of a voltage ramp of ± 50 mV in 1 M KCl and compared to the signal of the bilayer membrane without any addition (A).

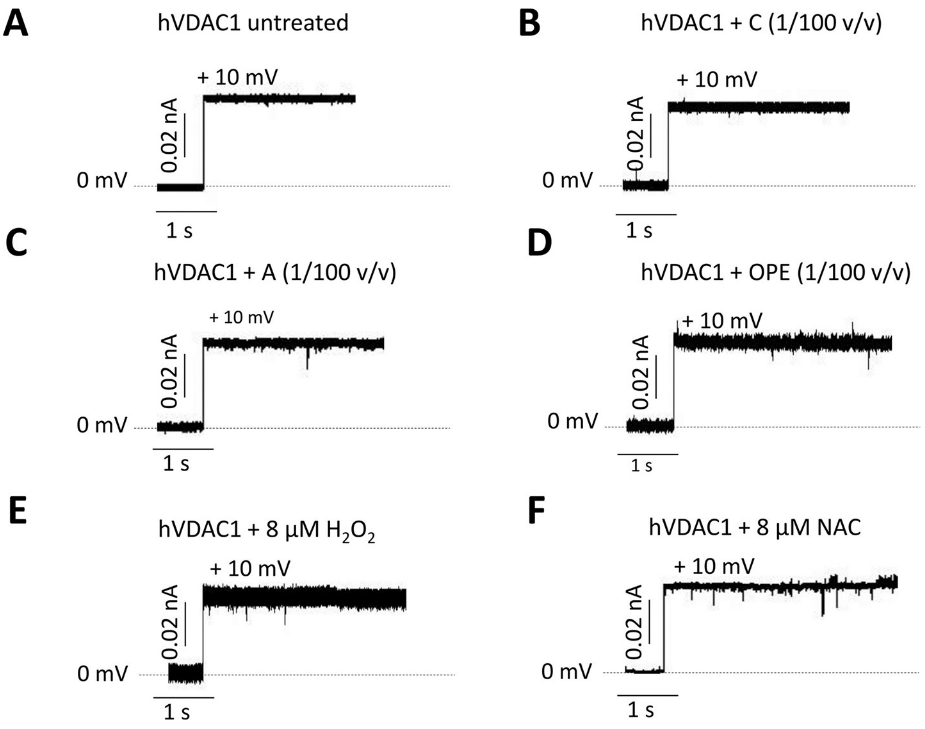

Figure S4. Single channel recordings of hVDAC1 in presence of tested samples, hydrogen peroxide and N-acetyl cysteine (NAC). Representative current traces registered upon no addition (A) or addition of samples A (B), C (C), and OPE (D), H2O2 (E) and NAC (F) to both sides of a DiphPC bilayer containing hVDAC1 at a final concentration of 1:100 (v/v) and 8 µM respectively. Current measurements were performed in symmetrical 1 M KCl at + 10 mV applied.
